# Modification Effect of *PARP4* and *ERCC1* Gene Polymorphisms on the Relationship between Particulate Matter Exposure and Fasting Glucose Level

**DOI:** 10.3390/ijerph19106241

**Published:** 2022-05-20

**Authors:** Jin Hee Kim, Seungho Lee, Yun-Chul Hong

**Affiliations:** 1 Department of Integrative Bioscience & Biotechnology, Sejong University, 209 Neungdong-ro, Gwangjin-gu, Seoul 05006, Korea; 2 Department of Preventive Medicine, College of Medicine, Dong-A University, 32 Daesingongwon-ro, Seo-gu, Busan 49201, Korea; seunglee@dau.ac.kr; 3 Department of Preventive Medicine, Seoul National University College of Medicine, 28 Yongon-dong, Chongno-gu, Seoul 03080, Korea; ychong1@snu.ac.kr

**Keywords:** particulate matter, PM_10_, PM_2.5__–10_, PM_2.5_, fasting glucose, repair gene, *PARP4*, *ERCC1*, genetic polymorphism

## Abstract

Particulate matter (PM) has been linked to adverse health outcomes, including insulin resistance (IR). To evaluate the relationships between exposures to PM_10_, PM_2.5–10_, and PM_2.5_; the serum level of fasting glucose, a key IR indicator; and effects of polymorphisms of two repair genes (*PARP4* and *ERCC1*) on these relations, PMs exposure data and blood samples for glucose measurement and genotyping were collected from 527 Korean elders. Daily average levels of PMs during 8 days, from 7 days before examination to the health examination day (from lag day 7 to lag day 0), were used for association analyses, and mean concentrations of PM_10_, PM_2.5–10_, and PM_2.5_ during the study period were 43.4 µg/m^3^, 19.9 µg/m^3^, and 23.6 µg/m^3^, respectively. All three PMs on lag day 4 (mean, 44.5 µg/m^3^ for PM_10_, 19.9 µg/m^3^ for PM_2.5–10_, and 24.3 µg/m^3^ for PM_2.5_) were most strongly associated with an increase in glucose level (percent change by inter-quartile range-change of PM: (β) = 1.4 and *p* = 0.0023 for PM_10_; β = 3.0 and *p* = 0.0010 for PM_2.5–10_; and β = 2.0 and *p* = 0.0134 for PM_2.5_). In particular, elders with *PARP4* G-C-G or *ERCC1* T-C haplotype were susceptible to PMs exposure in relation to glucose levels (*PARP4* G-C-G: β = 2.6 and *p* = 0.0006 for PM_10_, β = 3.5 and *p* = 0.0009 for PM_2.5–10_, and β = 1.6 and *p* = 0.0020 for PM_2.5_; *ERCC1* T-C: β = 2.2 and *p* = 0.0016 for PM_10_, β = 3.5 and *p* = 0.0003 for PM_2.5–10_, and β = 1.2 and *p* = 0.0158 for PM_2.5_). Our results indicated that genetic polymorphisms of *PARP4* and *ERCC1* could modify the relationship between PMs exposure and fasting glucose level in the elderly.

## 1. Introduction

Insulin resistance (IR), a prediabetic state of type 2 diabetes, is an important health issue with a rapidly increasing incidence worldwide [1,2]. Several studies reported positive associations between particulate matters (PMs) and serum levels of fasting glucose, an important IR indicator [3,4]. The adverse effect of PM_10_ with a diameter of less than 10 μm on fasting glucose level was proven in the general population, regardless of short-term or long-term exposure [3,4]. Although short-term or long-term exposure to PM_2.5_ with a diameter of less than 2.5 μm was also reported to induce IR in C57BL/6 mice and incident metabolic syndrome in KORA cohort [5,6], evidence for the effects of PM_2.5_ and particularly PM_2.5–10_, known as coarse PM, on fasting glucose levels was insufficient.

Oxidative stress is considered to be a major biological mechanism underlying IR [7]. Although a variety of potential mechanisms of PMs inducing IR have been suggested, oxidative stress is still at the center of research attention [8,9,10,11]. Exposure to PM_2.5_ in mice can cause vascular IR by inducing pulmonary oxidative stress [8,12]. Furthermore, the adverse effect of PM_2.5_ on vascular IR can be reduced by removing superoxide from the lungs of mice [11,12]. The water-insoluble fraction of PM_10_ can also induce oxidative stress in human lung epithelial A549 cells [13]. Because PMs contain pro-oxidant molecules, such as chromium, iron, nickel, and zinc, that can induce reactive oxygen species [10], oxidative stress is plausible as a major mechanism of PMs on IR. Because oxidative stress could induce oxidative DNA damages, cells need to up-regulate DNA repair genes to protect against oxidative DNA damage upon PMs exposure [14]. In fact, an epidemiologic study reported that PMs exposure could even induce oxidative stress-associated DNA damage in healthy young adults exposed to low concentrations of ambient PM_2.5–10_ and PM_2.5_ [15].

Poly(ADP-ribose) polymerase family member 4 (PARP4) and excision repair cross-complementation 1 (ERCC1), respectively, repair DNA damage using base excision and nucleotide excision repair pathways to remove oxidized DNA bases or nucleotides [16,17]. These DNA damage repairs are considered to be very important processes because they can protect the human body against oxidative stress. It has been reported that PARP is activated by DNA strand breakage through the excessive accumulation of reactive oxygen species in relation to hyperglycemia [18]. Although *PARP1* and *PARP2*, included in the same family as *PARP4*, were reported to affect glucose metabolism and insulin sensitivity [19], there is no evidence for the effect of *PARP4* on blood glucose level. On the other hand, *ERCC1* gene was reported to have an impact on glucose intolerance in a progeroid mouse model with ERCC1 deficiency, resulting in fat loss and IR by triggering an autoinflammatory response [20]. Previous evidence showed that genetic variations of *PARP4* and *ERCC1* could potentially affect IR differently by changing the capacity of corresponding enzymes encoded by them.

As population aging increases, we need to pay attention to the elderly, who are more vulnerable to chronic diseases than others [21]. With increasing age, the body’s function as well as immune system become more sensitive and vulnerable [21]. The EpiAir study, an epidemiologic surveillance on air pollution and Italian health, indicated that elderly subjects were more vulnerable to exposure to particulate matters than to other pollutants [21]. Therefore, the objective of the present study was to evaluate relations between PMs (PM_10_, PM_2.5__–__10_, and PM_2.5_) exposure and the serum level of fasting glucose among the elderly population considering modification by genetic polymorphisms of *PARP4* and *ERCC1*.

## 2. Materials and Methods

### 2.1. Study Population and Sampling

The Korean Elderly Environmental Panel I study began from 2008. There were five repeated health examinations from start time to 2010 (twice in 2008, once in 2009, and twice in 2010) for 560 participants aged 60 or over recruited at a community welfare center for elders in Seoul, Korea. After excluding participants lacking blood samples and PM concentrations data, the final analysis included 527 participants. We asked participants to fast from midnight on the day before the examination. We collected their blood samples on the day of visit at around 10 A.M. Whole blood was centrifuged at 30 to 60 min after collection, and the serum and cellular layer were separated and stored in screw-cap tubes. All serum samples were frozen at −70 °C until analysis for glucose level measurement. The cellular layer was also stored at −70 °C for DNA preparation. To analyze urinary cotinine levels, spot urine samples were also collected and then stored at −20 °C until analysis. Information about basic demographics, including smoking status, was collected through an interview by trained staff.

### 2.2. PM Concentrations and Meteorological Factors

PM_10_ and PM_2.5_ concentrations were collected from the Korea National Institute of Environmental Research and Seoul Research Institute of Public Health and Environment, respectively, as day average levels during 8 days from seven days before to the health examination day (from lag day 7 to lag day 0) [4,22]. PM_2.5__–__10_ concentration was calculated as the difference between PM_10_ and PM_2.5_ concentrations. Daily outdoor temperature and dewpoint of the day were obtained from the Korea Meteorological Administration. All PM concentrations and meteorological data were obtained from monitoring sites nearest to the residence of participants. Daily means were calculated and used as individual values corresponding to each participant.

### 2.3. Fasting Glucose Level Measurement

Serum level of fasting glucose was measured using a hexokinase method with a Pureauto S GLU kit (Daiichi Pure Chemicals, Tokyo, Japan) [4].

### 2.4. Cotinine Measurement

To determine exposure to tobacco smoke, urinary cotinine level was measured using an enzyme-linked immunosorbent assay (Cotinine Elisa; Bio-Quant, San Diego, CA, USA) following the manufacturer’s procedure [4]. The lower limit of detection (LOD_L_) of cotinine level was 1 µg/g, and the value under LOD_L_ was assigned as 0.5 µg/g. A value greater than the upper LOD (LOD_U_ = 10,000 µg/g) was assigned as 15,000 µg/g. Cotinine level was adjusted for urinary creatinine level in statistical analyses.

### 2.5. Genotyping

We listed all single-nucleotide polymorphisms (SNPs) on *PARP4* and *ERCC1* and examined their minor allele frequencies in Asian population using International HapMap data because low minor allele frequency may lead to null result; although, the SNP is meaningful for the risk of target outcome. After we searched for linkage structure using the Haploview to confirm which SNPs should be selected for haplotype construction, we finally selected three SNPs of *PARP4* (rs12863638, rs3783073, and rs2275660) and two SNPs of *ERCC1* (rs11615 and rs3212961) (Table A1). Table A1 shows detailed information for these five SNPs including rs number, Human Genome Variation Society (HGVS) name, chromosome number, their position, function, call rate, and accuracy. All five SNPs were genotyped using TaqMan method. In brief, a polymerase chain reaction (PCR) was carried out with a final volume of 5 μL, containing 10 ng of genomic DNA, 2.5 μL of 2X TaqMan Universal PCR Master Mix, 0.125 μL of 40X Assay Mix, and 1.25 μL of DNase-free water (Assay ID, C_9228399_10 for rs12863638; C_27515784_10 for rs3783073; C_15879320_10 for rs2275660; C_2532959_1 for rs11615; and C_25934767_10 for rs3212961). Thermal cycle conditions were: 50 °C for 2 min and 95 °C for 10 min, followed by 45 cycles at 95 °C for 15 s and 60 °C for 1 min. Dual 384-Well GeneAmp PCR System 9700 (ABI, Foster City, CA, USA) was used for PCR. Endpoint fluorescent readings were conducted using an ABI PRISM 7900 HT Sequence Detection System (ABI, Foster City, CA, USA). Five percent of samples were randomly chosen for repeated testing. Identical results were found with 100% accuracy rate (Table A1). We also tested the Hardy–Weinberg Equilibrium (HWE) for genotyped SNPs using chi-square test and found that all *p*-values were larger than 0.05, indicating that all SNPs were in HWE.

### 2.6. Haplotype Determination

Because adjacent SNPs in the same chromosome region can be inherited together in a haplotype, an analysis using multiple SNPs can simultaneously increase statistical sensitivity [23] or the power to detect loci relative to that of single SNPs [24]. Therefore, haplotypes composed of SNPs of each gene were made using PHASE program version 2.1 (http://stephenslab.uchicago.edu/phase/download.html, accessed on 14 May 2018). In brief, we deleted the data of people whose genotype was lacking for at least one SNP, and then composed haplotypes based on three SNPs of *PARP4* (rs12863638, rs3783073, and rs2275660) and two SNPs of *ERCC1* (rs11615 and rs3212961). The linkage disequilibrium (LD) between two SNPs of each gene was evaluated based on relative disequilibrium (D′) [25]. Statistical significance of LD was evaluated using Fisher’s exact test.

### 2.7. Statistical Analysis

Baseline characteristics of participants were compared between males and females. Student’s t-test was used for mean comparison and chi-square test was used for frequency comparison. Mean concentrations and selected percentiles of repeated measures, including fasting glucose and PMs, were calculated. For PMs, the day average from lag day 0 to lag day 7 was used. Original or log-transformed concentrations of PM_10_, PM_2.5__–10_, and PM_2.5_ were compared among lag days using analysis of variance (ANOVA). Relations among PM_10_, PM_2.5__–__10_, and PM_2.5_ levels on each lag day were evaluated using Pearson’s correlation. After fasting glucose levels were log-transformed for their normality, the relation of each PM with glucose level was estimated using mixed effect models since we repeated measurements for both PMs and glucose level. The relation of each PM with glucose level was also evaluated by genotypes and diplotypes of *PARP4* and *ERCC1* genes. In all models, changes in glucose level by inter-quartile range (IQR) changes in PM_10_, PM_2.5__–__10_, and PM_2.5_ were evaluated after adjusting for age (year), sex (males vs. female), body mass index (BMI, kg/m^2^), urinary cotinine level (μg/g creatinine), outdoor temperature (°C), and dewpoint (°C) of the day. Statistical significance was considered when *p*-value was lower than 0.05. All statistical analyses were performed using SAS version 9.4 (SAS Institute Inc., Cary, NC, USA).

## 3. Results

### 3.1. Baseline Characteristics of Study Participants

The study participants were 527 elders (Table 1). There were 136 (25.8%) males and 391 (74.2%) females, and the average visit number was 3.3. The mean age was 70.8 years for all participants, and most participants (n = 294, 55.8%) had BMI < 25. Frequencies of five SNPs were not significantly different between males and females (*p* > 0.05 for all five) (Table 1).

### 3.2. Distribution of Repeated Measures during the Study Period

The average fasting glucose level was 5.5 mmol/L during the study period, while the mean concentrations of PM_10_, PM_2.5–10_, and PM_2.5_ were 43.4 µg/m^3^, 19.9 µg/m^3^, and 23.6 µg/m^3^, respectively (Table 2). The mean outdoor temperature and dewpoint of the day were 17.2 °C and 6.1 °C, respectively (Table 2). The IQR was 22.7 µg/m^3^ for PM_10_, 16.2 µg/m^3^ for PM_2.5–10_, and 9.0 µg/m^3^ for PM_2.5_ (Table 2).

### 3.3. PM Concentrations and Their Correlations

**Figure 1:**
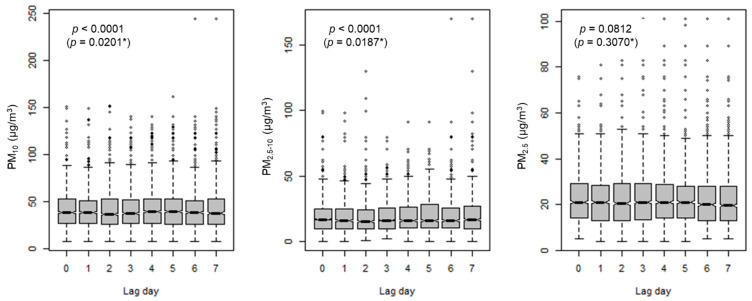
PMs exposure by lag days. Original or * log-transformed concentrations of PM_10_, PM_2.5__–10_, and PM_2.5_ were compared among lag days using ANOVA. ANOVA, analysis of variance.

In the comparison of PM_10_, PM_2.5–10_, and PM_2.5_ concentrations among lag days, PM_10_ and PM_2.5–10_ concentrations were significantly different among lag days for both original and log-transformed concentrations (*p* < 0.05 for all), while PM_2.5_ concentrations were not significantly different among lag days; although, original values showed marginal significance (*p* = 0.0812) (Figure 1). However, each PM_10_ or PM_2.5–10_ concentration was correlated among lag days (all *p* < 0.0001 with correlation coefficients ranging from 0.14 to 0.72 for PM_10_ and from 0.24 to 0.66 for PM_2.5–10_), while PM_2.5_ concentration showed no significant correlations between levels on lag day 0 and levels on lag days 4 to 7 and between levels on lag day 1 and levels on lag days 6 and 7, although levels on other lag days were significantly correlated with each other (Table A2).

Since PM_2.5–10_ and PM_2.5_ were included in PM_10_, relations among PMs on the same day were also evaluated (Table A2). All three PMs were strongly correlated with each other with the maximum and minimum correlation coefficients of 0.92 and 0.54 (all *p* < 0.0001) (Table A2).

### 3.4. Association between PMs Exposure and Fasting Glucose Level

Relations between PM concentrations on eight lag days (from lag day 0 to lag day 7) and fasting glucose level were evaluated (Table 3). All three PMs significantly or marginally increased glucose levels from lag days 3 to 7, with the strongest effect on lag day 4, with mean concentrations of 44.5 µg/m^3^ for PM_10_, 19.9 µg/m^3^ for PM_2.5–10_, and 24.3 µg/m^3^ for PM_2.5_ (percent change by IQR; change in PM: (β) = 1.4 and *p* = 0.0023 for PM_10_; β = 3.0 and *p* = 0.0010 for PM_2.5–10_; and β = 2.0 and *p* = 0.0134 for PM_2.5_) (Table 3). Because PMs on lag day 4 showed the strongest associations with glucose level based on the effect size, lag day 4 was chosen for further analyses.

### 3.5. Association between PMs Exposure and Fasting Glucose Level by Genotypes or Diplotypes of Repair Genes

Relation between PMs exposure and glucose level was evaluated by genotypes of *PARP4* and *ERCC1*. Participants with G allele for rs12863638, C allele for rs3783073, G allele for rs2275660, T allele for rs11615, and C allele for rs3212961 seemed to be susceptible to exposure to three PMs in relation to the increase in glucose level, although the *ERCC1* TT genotype for rs11615 was marginally significant with the greatest effect size (Table 4).

All three SNPs of *PARP4* and two SNPs of *ERCC1* were in a strong LD with each other (*p* < 0.0001 for all relations). For *PARP4*, D′ was 0.95 between rs12863638 and rs3783073, 0.71 between rs12863638 and rs2275660, and 0.98 between rs3783073 and rs2275660. For *ERCC1*, D′ between rs11615 and rs3212961 was 0.89. Because the G allele for rs12863638, C allele for rs3783073, and G allele for rs2275660 of *PARP4* and T allele for rs11615 and C allele for rs3212961 of *ERCC1* showed an increasing trend for the susceptibility to PMs exposure and there were strong linkages among SNPs of same gene, we evaluated the relationship between PMs exposure and glucose level by combined diplotypes of *PARP4* and *ERCC1*. First of all, we created the risky haplotypes of *PARP4* (G-C-G haplotype composed of G allele for rs12863638, C allele for rs3783073, and G allele for rs2275660) and *ERCC1* (T-C haplotype composed of T allele for rs11615 and C allele for rs3212961) and evaluated the effect of PMs exposure on glucose level in participants with or without risky haplotype (Table 4). For all three PMs, participants with the *PARP4* G-C-G haplotype or *ERCC1* T-C haplotype were found to be susceptible to PMs exposure based on their glucose levels (*PARP4* G-C-G: β = 2.6 and *p* = 0.0006 for PM_10_, β = 3.5 and *p* = 0.0009 for PM_2.5–10_, and β = 1.6 and *p* = 0.0020 for PM_2.5_; *ERCC1* T-C: β = 2.2 and *p* = 0.0016 for PM_10_, β = 3.5 and *p* = 0.0003 for PM_2.5–10_, and β = 1.2 and *p* = 0.0158 for PM_2.5_), while others (participants without *PARP4* G-C-G haplotype or *ERCC1* T-C haplotype) were not susceptible (*p* > 0.05 for all three PMs and both genes) (Table 4).

We also evaluated the interaction between PMs exposure and genotypes or diplotypes in relation to glucose level (Table 4). Two PMs (PM_10_ and PM_2.5_) showed significant interactions with *PARP4* diplotypes for glucose level (*p* = 0.0262 for PM_10_ and *p* = 0.0191 for PM_2.5_), while PM_2.5_ showed marginal significant interactions with *PARP4* diplotypes (*p* = 0.0802) (Table 4). However, *ERCC1* diplotypes did not show interactions with all three PMs for glucose level (*p* > 0.05 for all three PMs) (Table 4).

We also evaluated direct effects of genes on glucose level, but did not find significant effects of genes on glucose level (*p* > 0.05 for both genes) (Table A3).

## 4. Discussion

In the present study, all three PMs were strongly correlated with each other and PM_2.5_ had a higher daily variability than the other PMs. Furthermore, participants with *PARP4* G-C-G and *ERCC1* T-C haplotypes were susceptible to PMs exposure in relation to fasting glucose level.

The World Health Organization (WHO) has suggested a daily average level of 45 µg/m^3^ for PM_10_ and 15 µg/m^3^ for PM_2.5_ in their Global Air Quality Guidelines [26]. Compared to the Global Air Quality Guidelines by WHO, our elderly population were exposed to a relatively and consistently high level of PMs, particularly PM_2.5_. Considering correlations among three PMs on a specific lag day or among several lag days for the same PM in our study, PMs were temporally variable, particularly PM_2.5_, although their levels were related to each other. In the present study, we chose lag day 4 for further analyses based on the strongest associations of PMs on lag day 4 with glucose level in the present study, and found the biggest change in glucose level after PM_2.5–10_ exposure, followed by PM_2.5_ and PM_10_. The biggest change in glucose level after PM_2.5–10_ exposure was supported by previously reported studies. Liang et al. [27] showed that each increase of 10 µg/m^3^ in 3-day moving averages of PM significantly increased the risk of outpatient visits of pneumonia, bronchiolitis, and asthma, regardless of PM size, with PM_2.5–10_ showing the biggest effect size (4.36% of PM_10_, 9.19% of PM_2.5–10_, and 3.71% of PM_2.5_ for outpatient visits of pneumonia; 3.12% of PM_10_, 9.13% of PM_2.5–10_, and 3.21% of PM_2.5_ for bronchiolitis; and 3.33% of PM_10_, 11.69% of PM_2.5–10_, and 3.45% of PM_2.5_ for asthma). Lei et al. [28] showed that PM_2.5–10_ had stronger associations with the loss of lung function than PM_2.5_. The greatest effect size for PM_2.5–10_ in our analyses could be attributable to the difference in the amount of lipopolysaccharide (LPS) among PMs. Biologic components intrinsic to PM such as LPS could directly activate Toll-like receptors, leading to inflammation [29]. In fact, LPS was reported to induce metabolic syndromes, such as IR [30,31]. Moreover, LPS was known to be dominant in urbanized environments in Asia [30]. The amount of LPS was reported to be higher in PM_10_ than in PM_2.5_ because of higher level of LPS in PM_2.5–10_ [30]. Because the effect size of PM_10_ could be offset by that of PM_2.5_ in our study, the effect size of PM_2.5–10_ on glucose level was the greatest. However, there is still a debate about which PM could play a role in oxidative-stress-related DNA damage. Feng et al. [32] indicated that DNA damage caused by fine particles, ranging from 0.43 to 2.1 μm in size, was greater than the damage caused by coarse particles, ranging from 4.7 to 10 μm in size. They also suggested that greater DNA damage in fine particles could be attributable to heavy metals enriched in fine particles [32]. Therefore, in the future, we need to clarify which PM size is more important for the prevention of oxidative-stress-related DNA damage as well as their biological functions and mechanisms.

DNA repair proteins such as PARP4 and ERCC1 can protect against metabolic dysfunction including IR [20,33]. Both PARP and ERCC family members were reported to be involved in the pathway of IR regulation through repairing oxidative DNA damage and inhibiting autoinflammatory response [20,33]. However, there is a lack of knowledge regarding the relation between PARP4, a particular member of PARP family, and fasting glucose level. Furthermore, the effect of *PARP4* and *ERCC1* variations on the relation between PMs exposure and glucose level has not yet been reported, although positive associations between DNA damage accumulation and the development of metabolic disorders were reported [33]. Because genetic polymorphisms of two repair genes, *PARP4* and *ERCC1*, can affect DNA repair efficiency [34], their polymorphisms may modify the relation between PMs exposure and glucose level. In the present study, we found that participants with *PARP4* G-C-G and *ERCC1* T-C haplotypes were apparently susceptible to an increase in glucose level in relation to exposure to all three PMs, although the effect size was slightly different by PMs. Although no study reported the effects of *PARP4* and *ERCC1* polymorphisms on relation between PMs exposure and fasting glucose level, several studies supported the potential idea that those polymorphisms could affect the impact of PMs exposure on glucose level. For the *ERCC1* gene, the defective *ERCC1* gene could increase the incidence of vascular diseases [35], and genetic polymorphisms of *ERCC1* could affect the efficiency of chemotherapy, as well as the susceptibility of a variety of diseases, including lung cancer and several cardiovascular diseases [36,37,38]. Particularly, the *ERCC1* rs11615 T allele was found to be a risk factor for developing non-small cell lung cancer [37]. Because the lungs can be directly exposed to PMs [8,11,12,13], the effects of these SNPs are plausible. In fact, the risk effect of rs11615 T allele [37] was consistent with our results, which we obtained with regard to both PMs and glucose level. Studies on *ERCC1* rs3212961 were controversial, although the rs3212961 C allele was associated with a shorter overall survival in gastric cancer patients [39] and a higher risk for non-Hodgkin lymphoma development [40], which were consistent with results of the present study. However, we found no evidence for the relation between the three SNPs of the *PARP4* gene examined in the present study and DNA damage or lung oxidation, although rs17080653, another intronic variant in *PARP4*, showed a protective effect on head and neck cancer [41] and inter-individual differences in DNA repair processes [42].

In the present study, we found interactions between PMs exposure and *PARP4* genotypes or diplotypes in relation to glucose level. All three PMs, in particular PM_10_ and PM_2.5_, showed interactions with *PARP4* diplotypes, although the direct effect of *PARP4* gene was not found. This meant the active functions of both environmental and genetic factors in relation with fasting glucose level, with an emphasis on the environmental factors in regulating genes. However, we did not find any significant interactions between PMs exposure and *ERCC1* diplotypes, although *ERCC1* rs11615 showed marginal interactions with PM_10_ and PM_2.5_ in relation with glucose. Therefore, in the future, we need to validate the interactive effect of the *ERCC1* gene with PMs in relation to glucose level with a larger sample size.

To the best of our knowledge, the present study is the first to explore the effects of genetic modifications of *PARP4* and *ERCC1* on relations between three PM species and fasting blood glucose level, targeting elders who are susceptible to environmental pollutants. Although we used a panel study design, which could increase statistical power, our study also had limitations. First, we did not control for other air pollutants (O_3_ or NO_2_) and *GST* family genes polymorphisms, although we found adverse effects of O_3_ and NO_2_ exposures on the glucose level and modification of *GSTM1* and *T1*, as well as an impact of *P1* polymorphisms on the relation between PM_10_ exposure and glucose level, in a previous study [4]. Because too many missing data could be produced if we matched O_3_ and NO_2_ to PM_10_ and PM_2.5_ on a daily basis, which could lead to non-significance due to small numbers of data used in the analyses, we did not control O_3_ or NO_2_ in our statistical models. This was the same for the *GSTM1*, *T1*, and *P1* genes. In the future, we need to confirm whether the modification effect of *PARP4* and *ERCC1* polymorphisms on the relations between PMs exposure and glucose level still remains after controlling for these factors with a larger sample size. Second, in the present study, we only explored the acute effects of PMs on glucose level. Therefore, we need to clarify whether our genetic modification effect still remains after long-term exposure and short-term exposure. Third, in the future, we need to consider the chemical nature of the particles to confirm the causality of particles as well as genes in relation to glucose level.

## 5. Conclusions

In conclusion, all three PMs were strongly correlated with each other and PM_2.5_ had higher daily variability than the others. Furthermore, elderly participants with *PARP4* G-C-G and *ERCC1* T-C haplotypes were susceptible to an increase in fasting glucose level after exposure to all three PMs, regardless of PM_10_, PM_2.5–10_, or PM_2.5_. These results should be confirmed in the future after considering other causal factors and confounders with a larger sample size.

## Figures and Tables

**Table 1:** Baseline characteristics of participants.

Characteristic	Total (N = 527)	Male (N = 136)	Female (N = 391)	*p*-Value
No. of visit, mean ± SE	3.3 ± 0.1	3.2 ± 0.1	3.4 ± 0.1	0.0710
Age, mean ± SE (range) (year)	70.8 ± 0.2 (60~87)	71.4 ± 0.4 (62~84)	70.6 ± 0.3 (60~87)	0.0812
Height, mean ± SE (cm)	154.7 ± 0.3	164.3 ± 0.4	151.4 ± 0.3	<0.0001
Weight, mean ± SE (kg)	59.4 ± 0.4	65.8 ± 0.8	57.1 ± 0.4	<0.0001
BMI, n (%) (kg/m^2^)				
≥30	22 (4.2)	5 (3.7)	17 (4.3)	0.5866
25~<30	211 (40.0)	50 (36.7)	161 (41.2)	
<25	294 (55.8)	81 (59.6)	213 (54.5)	
No. of current smokers, (%)	30 (5.7)	29 (21.3)	1 (0.3)	<0.0001
*PARP4* rs12863638, n (%)				
GG	238 (45.5)	63 (46.3)	175 (45.2)	0.3452
GT	238 (45.5)	57 (41.9)	181 (46.8)	
TT	47 (9.0)	16 (11.8)	31 (8.0)	
*PARP4* rs3783073, n (%)				
CC	214 (41.1)	61 (45.2)	153 (39.6)	0.2334
CT	245 (47.0)	63 (46.7)	182 (47.2)	
TT	62 (11.9)	11 (8.1)	51 (13.2)	
*PARP4* rs2275660, n (%)				
AA	231 (44.4)	50 (37.0)	181 (47.0)	0.0921
AG	222 (42.7)	68 (50.4)	154 (40.0)	
GG	67 (12.9)	17 (12.6)	50 (13.0)	
*ERCC1* rs11615, n (%)				
CC	291 (55.2)	74 (54.4)	217 (55.5)	0.9664
CT	203 (38.5)	53 (39.0)	150 (38.4)	
TT	33 (6.3)	9 (6.6)	24 (6.1)	
*ERCC1* rs3212961, n (%)				
CC	137 (26.4)	37 (27.2)	100 (51.1)	0.9334
CA	265 (51.2)	70 (51.5)	195 (51.1)	
AA	116 (22.4)	29 (21.3)	87 (22.8)	

BMI, body mass index; SE, standard error.

**Table 2:** Distribution of repeated measures during the study period.

			Selected Percentiles					
Repeated Measure	N	Mean (SE)	10th	25th	50th	75th	90th	95th
Urinary cotinine level, μg/g creatinine	1576	240.7 (34.7)	0.5	0.9	2.2	1.7	16.8	286.2
Fasting glucose level in serum, mmol/L	1065	5.5 (0.04)	4.6	4.8	5.2	5.8	6.7	7.4
PM_10_, μg/m^3^	1697	43.4 (0.4)	22.5	31.0	40.2	53.7	63.5	79.0
PM_2.5–10_, μg/m^3^	1697	19.9 (0.3)	8.6	11.2	16.7	27.4	33.4	41.1
PM_2.5_, μg/m^3^	1716	23.6 (0.2)	13.6	18.0	23.6	27.0	32.0	37.8
Outdoor temperature, °C	1752	16.8 (0.2)	3.0	11.0	18.2	24.3	26.2	26.7
Dewpoint, °C	1752	6.2 (0.2)	−8.3	−2.4	6.6	14.9	19.3	19.8

SE, standard error. Individual average concentrations of PM_10_, PM_2.5–10_, PM_2.5_, outdoor temperature and dewpoint from health examination day to lag day 7 were used for the calculation of mean and selected percentiles.

**Table 3:** Relations between PMs at each lag day and glucose level.

	PM_10_		PM_2.5__–10_		PM_2.5_	
Lag Day	% Change (95% CI)	*p*-Value	% Change (95% CI)	*p*-Value	% Change (95% CI)	*p*-Value
0	0.7 (−0.3, 1.6)	0.1727	1.7 (0.1, 3.2)	0.0343	0.4 (−1.5, 2.3)	0.6952
1	0.2 (−0.9, 1.2)	0.7658	−0.2 (−2.1, 1.7)	0.8192	0.8 (−1.1, 2.6)	0.4249
2	0.6 (−0.5, 1.7)	0.2742	1.0 (−0.9, 2.9)	0.3075	1.2 (-0.8, 3.2)	0.2396
3	1.2 (0.2, 2.2)	0.0191	1.9 (0.1, 3.8)	0.0422	1.8 (0.03, 3.5)	0.0464
4	1.4 (0.5, 2.3)	0.0023	3.0 (1.2, 4.8)	0.0010	2.0 (0.4, 3.5)	0.0134
5	0.8 (−0.03, 1.7)	0.0594	1.7 (0.03, 3.3)	0.0459	1.9 (0.2, 3.5)	0.0244
6	0.9 (0.1, 1.7)	0.0204	1.7 (0.4, 3.0)	0.0093	1.3 (−0.2, 2.9)	0.0976
7	1.2 (0.4, 2.0)	0.0032	2.4 (1.0, 3.7)	0.0005	1.6 (−0.1, 3.3)	0.0656

After glucose levels were log-transformed for their normality, percent changes in glucose levels by IQR-changes of PM_10_ (22.7 μg/m^3^), PM_2.5–10_ (16.2 μg/m^3^), and PM_2.5_ (9.0 μg/m^3^) were obtained after adjusted for age, sex, BMI, urinary cotinine level, and outdoor temperature and dewpoint of the day. BMI, body mass index; CI, confidence interval; IQR, inter-quartile range.

**Table 4:** Relations between PMs at lag day 4 and glucose level by genotypes or combined diplotypes of repair genes.

			PM_10_			PM_2.5–10_			PM_2.5_		
Gene	Genotype or Diplotype	No. (%)	% Change (95% CI)	*p*-Value	*p*-Value for Interaction	% Change(95% CI)	*p*-Value	*p*-Value for Interaction	% Change(95% CI)	*p*-Value	*p*-Value for Interaction
*PARP4*	rs12863638										
	GG	238 (45.5)	1.9 (0.4, 3.3)	0.0128	0.4411	2.9 (−0.1, 6.0)	0.0573	0.6577	3.4 (1.0, 5.8)	0.0064	0.1085
	GT	238 (45.5)	1.1 (−0.2, 2.3)	0.0964		3.4 (1.0, 5.8)	0.0055		0.5 (–1.7, 2.7)	0.6626	
	TT	47 (9.0)	−1.0 (−4.3, 2.3)	0.5639		−1.9 (−8.4, 4.6)	0.5770		–2.0 (–7.6, 3.5)	0.4755	
	rs3783073										
	CC	214 (41.1)	2.0 (0.5, 3.5)	0.0097	0.5046	4.4 (1.4, 7.3)	0.0041	0.4387	2.8 (0.1, 5.5)	0.0417	0.6745
	CT	245 (47.0)	1.2 (−0.2, 2.5)	0.0861		2.1 (−0.5, 4.8)	0.1174		1.9 (–0.4, 4.2)	0.1055	
	TT	62 (11.9)	0.6 (−1.3, 2.5)	0.5370		1.2 (–2.7, 5.2)	0.5377		0.8 (–2.2, 3.9)	0.5929	
	rs2275660										
	AA	231 (44.4)	0.6 (−0.6, 1.8)	0.3255	0.0129	1.5 (–0.9, 3.9)	0.2366	0.0853	0.6 (–1.3, 2.6)	0.5431	0.0033
	AG	222 (42.7)	1.6 (0.1, 3.0)	0.0389		3.7 (0.9, 6.5)	0.0099		1.8 (–0.8, 4.4)	0.1742	
	GG	67 (12.9)	5.1 (1.7, 8.4)	0.0042		9.1 (2.3, 15.9)	0.0113		8.8 (3.1, 14.6)	0.0038	
Without G-C-G haplotype		251 (49.0)	0.4 (–0.7, 1.6)	0.4647	0.0262	1.0 (–0.7, 2.7)	0.2485	0.0802	0.1 (–0.7, 0.9)	0.8277	0.0191
With G-C-G haplotype		261 (51.0)	2.6 (1.1, 4.0)	0.0006		3.5 (1.4, 5.5)	0.0009		1.6 (0.6, 2.6)	0.0020	
*ERCC1*	rs11615										
	CC	291 (55.2)	0.3 (−0.8, 1.5)	0.5781	0.0529	0.8 (–1.5, 3.2)	0.4874	0.1684	0.3 (–1.6, 2.3),	0.7410	0.0399
	CT	203 (38.5)	2.5 (1.0, 4.1)	0.0015		5.3 (2.3, 8.2)	0.0005		3.6 (0.8, 6.3)	0.0120	
	TT	33 (6.3)	3.1 (−0.2, 6.5)	0.0785		6.1 (–1.2, 13.3)	0.1147		5.2 (–0.4, 10.7)	0.0796	
	rs3212961										
	CC	137 (26.4)	2.7 (1.0, 4.5)	0.0026	0.3417	5.2 (1.8, 8.7)	0.0034	0.3733	4.2 (1.2, 7.1)	0.0069	0.2007
	CA	265 (51.2)	0.9 (−0.3, 2.2)	0.1348		2.8 (0.3, 5.3)	0.0282		0.9 (–1.2, 3.0)	0.4108	
	AA	116 (22.4)	1.2 (−1.0, 3.3)	0.2822		0.8 (–3.6, 5.1)	0.7301		2.7 (–0.9, 6.2)	0.1415	
Without T-C haplotype		295 (56.9)	0.9 (–0.3, 2.1)	0.1565	0.1746	1.2 (–0.6, 3.0)	0.1990	0.1548	0.5 (–0.3, 1.3)	0.1998	0.2609
With T-C haplotype		223 (43.1)	2.2 (0.9, 3.6)	0.0016		3.5 (1.6, 5.4)	0.0003		1.2 (0.2, 2.2)	0.0158	

After glucose levels were log transformed for their normality, percent changes in glucose levels by IQR changes in PM_10_ (22.7 μg/m^3^), PM_2.5–10_ (16.2 μg/m^3^), and PM_2.5_ (9.0 μg/m^3^) were obtained after adjusting for age, sex, BMI, urinary cotinine level, outdoor temperature and dewpoint of the day, by genotypes or combined diplotypes of repair genes. *p*-Value for interaction between PM exposure and genotypes or diplotypes was also evaluated. Each haplotype was composed of rs12863638, rs3783073, and rs2275660 for *PARP4,* and rs11615 and rs3212961 for *ERCC1*. BMI, body mass index; CI, confidence interval; IQR, inter-quartile range.

## Appendix A

**Table A1:** Genotyped polymorphisms of repair genes.

Gene	rs No.	HGVS Name	Chromosome No.	Position	Amino Acid Change	Call Rate (%)	Accuracy (%)
*PARP4*	rs12863638	c.-2+2528G>T	13	Intron in the 5′ UTR	-	99.2	100
	rs3783073	c.879+374T>C	13	Intron	-	98.8	100
	rs2275660	c.2695G>A	13	Codon 899	Ala899Thr	98.7	100
*ERCC1*	rs11615	c.354T>C	19	Codon 118	Asn118Asn	100	100
	rs3212961	c.525+33C>A	19	Intron	-	98.3	100

**Table A2:** Correlations among PM_10_, PM_2.5__–10_, and PM_2.5_ levels (Pearson correlation coefficients and *p*-values).

Pollutant					PM_10_								PM_2.5–10_								PM_2.5_				
	Lag Day	0	1	2	3	4	5	6	7	0	1	2	3	4	5	6	7	0	1	2	3	4	5	6	7
PM_10_	0	1	0.66	0.30	0.14	0.15	0.15	0.19	0.29	0.92	0.61	0.31	0.21	0.27	0.30	0.33	0.43	0.90	0.58	0.23	0.05	−0.03	−0.03	0.01	0.07
			<0.0001	<0.0001	<0.0001	<0.0001	<0.0001	<0.0001	<0.0001	<0.0001	<0.0001	<0.0001	<0.0001	<0.0001	<0.0001	<0.0001	<0.0001	<0.0001	<0.0001	<0.0001	0.0348	0.2653	0.1983	0.7519	0.0025
	1		1	0.66	0.34	0.22	0.25	0.19	0.23	0.58	0.90	0.57	0.34	0.28	0.32	0.25	0.33	0.61	0.90	0.60	0.28	0.08	0.11	−0.002	0.05
				<0.0001	<0.0001	<0.0001	<0.0001	<0.0001	<0.0001	<0.0001	<0.0001	<0.0001	<0.0001	<0.0001	<0.0001	<0.0001	<0.0001	<0.0001	<0.0001	<0.0001	<0.0001	0.0008	<0.0001	0.9338	0.0453
	2			1	0.66	0.39	0.30	0.28	0.22	0.27	0.59	0.88	0.59	0.39	0.33	0.29	0.27	0.26	0.59	0.88	0.59	0.29	0.21	0.12	0.07
					<0.0001	<0.0001	<0.0001	<0.0001	<0.0001	<0.0001	<0.0001	<0.0001	<0.0001	<0.0001	<0.0001	<0.0001	<0.0001	<0.0001	<0.0001	<0.0001	<0.0001	<0.0001	<0.0001	<0.0001	0.0028
	3				1	0.69	0.47	0.29	0.29	0.12	0.28	0.51	0.88	0.61	0.43	0.26	0.31	0.12	0.34	0.64	0.91	0.61	0.40	0.18	0.17
						<0.0001	<0.0001	<0.0001	<0.0001	<0.0001	<0.0001	<0.0001	<0.0001	<0.0001	<0.0001	<0.0001	<0.0001	<0.0001	<0.0001	<0.0001	<0.0001	<0.0001	<0.0001	<0.0001	<0.0001
	4					1	0.72	0.37	0.27	0.12	0.16	0.28	0.57	0.88	0.60	0.33	0.30	0.14	0.22	0.41	0.67	0.91	0.66	0.28	0.14
							<0.0001	<0.0001	<0.0001	<0.0001	<0.0001	<0.0001	<0.0001	<0.0001	<0.0001	<0.0001	<0.0001	<0.0001	<0.0001	<0.0001	<0.0001	<0.0001	<0.0001	<0.0001	<0.0001
	5						1	0.58	0.32	0.17	0.19	0.21	0.37	0.59	0.87	0.47	0.27	0.09	0.25	0.32	0.46	0.68	0.90	0.52	0.27
								<0.0001	<0.0001	<0.0001	<0.0001	<0.0001	<0.0001	<0.0001	<0.0001	<0.0001	<0.0001	00.0002	<0.0001	<0.0001	<0.0001	<0.0001	<0.0001	<0.0001	<0.0001
	6							1	0.63	0.23	0.20	0.27	0.28	0.35	0.51	0.91	0.54	0.10	0.13	0.21	0.24	0.33	0.52	0.89	0.55
									<0.0001	<0.0001	<0.0001	<0.0001	<0.0001	<0.0001	<0.0001	<0.0001	<0.0001	<0.0001	<0.0001	<0.0001	<0.0001	<0.0001	<0.0001	<0.0001	<0.0001
	7								1	0.32	0.25	0.23	0.34	0.34	0.31	0.57	0.91	0.20	0.16	0.15	0.21	0.17	0.26	0.55	0.84
										<0.0001	<0.0001	<0.0001	<0.0001	<0.0001	<0.0001	<0.0001	<0.0001	<0.0001	<0.0001	<0.0001	<0.0001	<0.0001	<0.0001	<0.0001	<0.0001
PM_2.5–10_	0									1	0.63	0.32	0.24	0.27	0.34	0.37	0.44	0.65	0.42	0.16	−0.01	−0.04	−0.03	0.05	0.11
											<0.0001	<0.0001	<0.0001	<0.0001	<0.0001	<0.0001	<0.0001	<0.0001	<0.0001		0.7571	0.0715	0.214	0.0662	<0.0001
	1										1	0.64	0.37	0.29	0.32	0.30	0.35	0.47	0.62	0.41	0.14	0.01	0.02	−0.004	0.06
												<0.0001	<0.0001	<0.0001	<0.0001	<0.0001	<0.0001	<0.0001	<0.0001	<0.0001	<0.0001	0.8108	0.4027	0.8733	0.0198
	2											1	0.61	0.38	0.32	0.36	0.31	0.23	0.39	0.55	0.33	0.13	0.06	0.11	0.07
													<0.0001	<0.0001	<0.0001	<0.0001	<0.0001	<0.0001	<0.0001	<0.0001	<0.0001	<0.0001	0.0144	<0.0001	0.0056
	3												1	0.66	0.46	0.34	0.39	0.13	0.23	0.42	0.61	0.38	0.22	0.13	0.15
														<0.0001	<0.0001	<0.0001	<0.0001	<0.0001	<0.0001	<0.0001	<0.0001	<0.0001	<0.0001	<0.0001	<0.0001
	4													1	0.65	0.42	0.46	0.21	0.21	0.30	0.45	0.60	0.40	0.17	0.09
															<0.0001	<0.0001	<0.0001	<0.0001	<0.0001	<0.0001	<0.0001	<0.0001	<0.0001	<0.0001	0.0003
	5														1	0.54	0.36	0.19	0.25	0.26	0.32	0.44	0.56	0.34	0.15
																<0.0001	<0.0001	<0.0001	<0.0001	<0.0001	<0.0001	<0.0001	<0.0001	<0.0001	<0.0001
	6															1	0.61	0.21	0.15	0.15	0.14	0.20	0.30	0.62	0.36
																	<0.0001	<0.0001	<0.0001	<0.0001	<0.0001	<0.0001	<0.0001	<0.0001	<0.0001
	7																1	0.33	0.24	0.16	0.18	0.11	0.14	0.36	0.54
																		<0.0001	<0.0001	<0.0001	<0.0001	<0.0001	<0.0001	<0.0001	<0.0001
PM_2.5_	0																	1	0.63	0.25	0.09	−0.02	−0.04	−0.05	0.01
																			<0.0001	<0.0001	0.0003	0.4303	0.1101	0.0589	0.6835
	1																		1	0.66	0.36	0.13	0.17	−0.01	0.02
																				<0.0001	<0.0001	<0.0001	<0.0001	0.5407	0.4813
	2																			1	0.71	0.37	0.31	0.10	0.05
																					<0.0001	<0.0001	<0.0001	<0.0001	0.0497
	3																				1	0.70	0.48	0.20	0.15
																						<0.0001	<0.0001	<0.0001	<0.0001
	4																					1	0.75	0.33	0.17
																							<0.0001	<0.0001	<0.0001
	5																						1	0.57	0.31
																								<0.0001	<0.0001
	6																							1	0.62
																									<0.0001
	7																								1
																									

**Table A3:** Direct effects of genotypes or combined diplotypes of repair genes on glucose level.

Gene	Genotype or Diplotype	No. (%)	% Change (95% CI)	*p*-Value	*p*-Value for Trend
*PARP4*	rs12863638				
	GG	238 (45.5)	Ref.		0.1456
	GT	238 (45.5)	–1.4 (–4.4, 1.6)	0.3489	
	TT	47 (9.0)	3.7 (–1.5, 8.8)	0.1671	
	rs3783073				
	CC	214 (41.1)	Ref.		0.2695
	CT	245 (47.0)	0.6 (–2.4, 3.7)	0.6969	
	TT	62 (11.9)	–3.3 (–8.1, 1.5)	0.1793	
	rs2275660				
	AA	231 (44.4)	Ref.		0.4948
	AG	222 (42.7)	0.2 (–2.9, 3.3)	0.9101	
	GG	67 (12.9)	2.7 (–1.9, 7.2)	0.2500	
Without G-C-G haplotype		251 (49.0)	Ref.		0.7902
With G-C-G haplotype		261 (51.0)	0.4 (–2.5, 3.3)	0.7902	
*ERCC1*	rs11615				
	CC	291 (55.2)	Ref.		0.2806
	CT	203 (38.5)	1.1 (–1.9, 4.1)	0.4855	
	TT	33 (6.3)	4.7 (–1.3, 10.8)	0.1222	
	rs3212961				
	CC	137 (26.4)	Ref.		0.5296
	CA	265 (51.2)	–1.9 (–5.3, 1.6)	0.2808	
	AA	116 (22.4)	–1.8 (–6.0, 2.3)	0.3883	
Without T-C haplotype		295 (56.9)	Ref.		0.5575
With T-C haplotype		223 (43.1)	0.9 (–2.0, 3.8)	0.5575	

After glucose levels were log-transformed for their normality, direct effect of genotypes or combined diplotypes of repair genes on glucose levels were evaluated after adjusting for age, sex, BMI, urinary cotinine level, outdoor temperature, and dewpoint of the day. Each haplotype was composed of rs12863638, rs3783073, and rs2275660 for *PARP4* and rs11615 and rs3212961 for *ERCC1*. BMI, body mass index; CI, confidence interval.
